# Early Life Irradiation-Induced Hypoplasia and Impairment of Neurogenesis in the Dentate Gyrus and Adult Depression Are Mediated by MicroRNA-34a-5p/T-Cell Intracytoplasmic Antigen-1 Pathway

**DOI:** 10.3390/cells10092476

**Published:** 2021-09-18

**Authors:** Hong Wang, Zhaowu Ma, Hongyuan Shen, Zijun Wu, Lian Liu, Boxu Ren, Peiyan Wong, Gautam Sethi, Feng Ru Tang

**Affiliations:** 1 Radiation Physiology Lab, Singapore Nuclear Research and Safety Initiative, National University of Singapore, Singapore 138602, Singapore; snrwh@nus.edu.sg (H.W.); snrsh@nus.edu.sg (H.S.); 2 The School of Basic Medicine, Health Science Center, Yangtze University, 1 Nanhuan Road, Jingzhou 434023, China; zhaowu823@126.com (Z.M.); zifanqie_00@126.com (L.L.); boxuren188@163.com (B.R.); 3 Huaxi MR Research Center (HMRRC), Functional and Molecular Imaging Key Laboratory of Sichuan Province, Department of Radiology, West China Hospital, Sichuan University, Chengdu 610017, China; 2019324020159@stu.scu.edu.cn; 4 Neuroscience Phenotyping Core, Department of Pharmacology, Yong Loo Lin School of Medicine, National University of Singapore, Singapore 117456, Singapore; wong_peiyan@nus.edu.sg; 5 Department of Pharmacology, Yong Loo Lin School of Medicine, National University of Singapore, Singapore 117600, Singapore

**Keywords:** γ-irradiation, depression, neurogenesis, miR-34a-5p, Tia1

## Abstract

Early life radiation exposure causes abnormal brain development, leading to adult depression. However, few studies have been conducted to explore pre- or post-natal irradiation-induced depression-related neuropathological changes. Relevant molecular mechanisms are also poorly understood. We induced adult depression by irradiation of mice at postnatal day 3 (P3) to reveal hippocampal neuropathological changes and investigate their molecular mechanism, focusing on MicroRNA (miR) and its target mRNA and protein. P3 mice were irradiated by γ-rays with 5Gy, and euthanized at 1, 7 and 120 days after irradiation. A behavioral test was conducted before the animals were euthanized at 120 days after irradiation. The animal brains were used for different studies including immunohistochemistry, CAP-miRSeq, Real-Time Quantitative Reverse Transcription PCR (qRT-PCR) and western blotting. The interaction of miR-34a-5p and its target T-cell intracytoplasmic antigen-1 (Tia1) was confirmed by luciferase reporter assay. Overexpression of Tia1 in a neural stem cell (NSC) model was used to further validate findings from the mouse model. Irradiation with 5 Gy at P3 induced depression in adult mice. Animal hippocampal pathological changes included hypoplasia of the infrapyramidal blade of the stratum granulosum, aberrant and impaired cell division, and neurogenesis in the dentate gyrus. At the molecular level, upregulation of miR-34a-5p and downregulation of Tia1 mRNA were observed in both animal and neural stem cell models. The luciferase reporter assay and gene transfection studies further confirmed a direct interaction between miR-34a-5p and Tia1. Our results indicate that the early life γ-radiation-activated miR-34a-5p/Tia1 pathway is involved in the pathogenesis of adult depression. This novel finding may provide a new therapeutic target by inhibiting the miR-34a-5p/Tia1 pathway to prevent radiation-induced pathogenesis of depression.

## 1. Introduction

Depression is one of the most significant and long-term consequence of radiation exposure among the survivors of nuclear accidents or wars [1,2,3,4] and is associated with brain-structural changes including reduced dentate gyrus size and altered hippocampal volume [5,6,7,8,9,10,11,12]. The small dentate gyrus may be caused by the impairment of neurogenesis, and it supports the “neurogenesis hypothesis of depression” [13] and the “cellular plasticity hypothesis of depression” [14]. The therapeutic effect achieved by promoting neurogenesis to improve depression symptoms further supports the “neurogenesis hypothesis of depression” [11,15,16]. These neuropathological changes may be induced by pre- or post-natal radiation exposure [17,18,19,20,21,22,23]. While it has been well documented that oxidative stress and neuroinflammation are involved in radiation-induced brain damage, whether brain microRNA (miR) and its target gene are involved in radiation-induced hippocampal structural changes and subsequent depression remains unknown.

Recent studies suggest that brain miR changes may be involved in depression-like behavior or depressive symptoms [24,25,26,27,28]. For instance, miR-15b is upregulated in the medial prefrontal cortex of mice with depression-like behavior and inhibits neuronal progenitor proliferation [29]. miR-124 serves as a putative therapeutic target and a biomarker for depression [30], and the inhibition of miR-124 could reduce depression-like behavior in animals [31,32,33]. miR-34a was shown to be significantly up-regulated in a mouse model [34] and in patients [35] with depression. In the latter, the serum level of miR-34a-5p was positively correlated with the severity of depression [35]. It suggests that further study of brain miR-34a-5p and its target gene may be needed in order to correlate serum miR-34a-5p changes to brain changes. Establishment of the relationship between brain miR-34a-5p, its target gene and the impairment of neurogenesis or small dentate gyrus may provide new clues for understanding the mechanism of the development of depression and for novel therapeutic approaches to prevent the genesis of depression.

The formation of stress granules (SGs) is a key event in cells after exposure to physiological or environmental stressors. The suppression of SG generation may underlie the neuronal cell death observed in neurodegenerative diseases [36]. T-cell intracytoplasmic antigen-1 (Tia1), as an RNA-binding protein in brain tissues, functions as a posttranscriptional regulator of gene expression. It aggregates to form SG following cellular damage, which is strongly linked to the pathophysiology of neurodegeneration [37,38]. Evidence has demonstrated that Tia1 is a potential biomarker in the brain of a mouse model for Alzheimer′s disease [39]. One study predicts that miR-34a may target Tia1 in the inhibition of myeloid-derived suppressor cell apoptosis [40]. However, the function of Tia1 on gamma irradiation-induced cellular damage has been poorly investigated.

This study aimed to examine if the acute irradiation with 5Gy at postnatal day 3 (P3) induced adult depression. The progressive neuropathological changes in the dentate gyrus, in particular, neurogenesis at 1, 7, and 120 day(s) after radiation exposure, were also investigated. We also elucidated the molecular mechanisms involved in radiation-induced neuropathological and neuropsychological changes, focusing on the miR and its targeted gene. We found that the γ-irradiation-activated miR-34a-5p/T-cell intracytoplasmic antigen-1 (Tia1) pathway in P3 mice was involved in the hypoplasia of the infrapyramidal blade of the stratum granulosum and the impairment of neurogenesis in the dentate gyrus and, therefore, participated in the pathogenesis of adult depression.

## 2. Materials and Methods

### 2.1. Radiation Exposure

Balb/c mice were purchased from InVivos Pte. Ltd. (Singapore) and housed with free access to water and food in the Comparative Medicine Facility, National University of Singapore. The experimental protocols were approved by the Institutional Animal Care and Use Committee (IACUC), National University of Singapore (IACUC number: R15-1576). Mice were exposed to 5 Gy gamma radiation (dose rate: 3.33 Gy/m) in a γ-Irradiator (BIOBEAM GM 8000, The Gamma-Service Medical GmbH, Leipzig, Germany) at postnatal day 3 (P3). Animals were euthanized at 1, 7 and 120 day(s) after irradiation and brain samples were collected for different experimental studies. For animals kept for 120 days, different neurobehavioral tests were performed before the animals were euthanized.

### 2.2. Behavioral Tests

#### 2.2.1. Open Field (Locomotor) Test

Mice were allowed to explore freely for 1 h in a square open field (40 cm × 40 cm) cage. Locomotor activity in terms of total distance travelled was recorded using the TopScan Behavioural Analysing system (Cleversys, Reston, VA, USA).

#### 2.2.2. Tail Suspension Test

Mice were suspended by their tails for 6 min [41]. Immobility time was detected by a strain gauge in the Tail Suspension Chamber (Med Associates Inc., St. Albans, VT, USA). A duration of time in which the force of the mouse′s movement did not exceed a set threshold was counted as immobility time.

#### 2.2.3. Forced Swim Test

The mice were put inside a cylinder filled with water for 6 min. The movement of the animal was recorded and analyzed using ForcedSwimScan (Cleversys, Reston, VA, USA). Floating time (during which the animal remained almost immobile and with its head above water) was used as a parameter to indicate depression-like behavior.

### 2.3. Immunohistochemical Staining

Animals were anaesthetized at 1, 7 and 120 day(s) post-irradiation. After perfusion with 4% paraformaldehyde, brain tissues were dissected, postfixed and then kept in 30% sucrose in 0.1 M phosphate buffer (pH: 7.4). Sagittal brain sections were cut at 40 μm and processed for Ki67, NeuN and doublecortin (DCX) immunohistochemistry in free floating sections. After treatment with 3% H_2_O_2_ and blocking with serum, free-floating sections were incubated with antibodies against DCX (1:500; Santa Cruz Biotechnology Inc., Santa Cruz, CA, USA), NeuN (1:500; Gene Tex, Hsinchu City, Taiwan), and Ki 67 (1:400; Gene Tex, Hsinchu City, Taiwan) overnight. The sections were then washed and incubated with respective secondary antibodies followed by avidin–biotin complex (ABC) reagent (Vector Laboratories Inc., Burlingame, CA, USA). After reaction in DAB Peroxidase Substrate (Vector Laboratories Inc., Burlingame, CA, USA), the sections were washed, mounted, counterstained and covered. The slides were examined and photographed under microscopy (Leica Microsystems GmbH, Wetzlar, Germany). The Stereologer System (Stereology Resource Center, Biosciences Inc. Tampa, FL, USA) was used to unbiasedly analyze the number of DCX and Ki67 immunopositive cells in the subgranular zone, and indicated the number/volume of the hilus of the dentate gyrus (mm^3^).

### 2.4. RNA Extraction from the Mouse Brain

RNA extraction from brain samples was performed using the miRNeasy Mini Kit (Qiagen, Hilden, Germany). The cerebrum was homogenized, and RNA extraction was performed according to the manufacturer′s instructions. RNA concentration and integrity were checked using the Nanodrop and Bioanalyzer system (Agilent Technologies, Santa Clara, CA, USA) before being subjected to miR sequencing and qPCR analysis.

### 2.5. Systematic miR Sequencing (miRSeq) Analysis

miRSeq was carried out using CAP-miRSeq (Molecular Genomics Pte Ltd., Singapore). The detected miRs were further compared between the control and irradiated animals and summarized using a heatmap.

### 2.6. Real-Time Quantitative Reverse Transcription PCR (qRT-PCR) Analysis of miR 

RNA was first reversely transcribed using the miScript II RT kit (Qiagen, Hilden, Germany). The 20 µL reaction mixture, including 4 µL 5× HiSpec buffer, 2 µL 10× nucleotide mix, 2 µL reverse transcripts mix, 5 µL template RNA and 7 µL nuclease-free water, was incubated at 37 °C for 1 h followed by 95 °C for 5 min. The resulting cDNA was then diluted by adding 80 µL of nuclease-free water and stored at −80 °C. 

Twenty microliters of master mix, for real time PCR, was prepared as follows: 2 µL diluted cDNA, 10 µL 2× miScript SYBR green PCR master mix, 2 µL 10× miScript universal primer and 2 µL primer for target miR, and 4 µL nuclease-free water. Samples were denatured at 95 °C for 15 min, followed by 40 cycles of denaturation at 94 °C for 15 sec, annealing at 55 °C for 30 sec, and extension at 70 °C for 30 s PCR amplification was carried out in QuantStudio 6 Real-Time PCR Systems (Thermo Fisher Scientific, Waltham, MA, USA). Fluorescence data were then collected. The average expression of miR-68 and miR-64 was used as internal control.

### 2.7. Predication of miR-34a-5p Targets and Luciferase Reporter Assay

Several online databases (TargetScan, miRanda, TarBase, miR2Disease, miRTarBase, miRecords, miRWalk) were used to analyze and predict the potential target genes of miR-34a-5p, one of the miRs that shows significant changes after irradiation. Tia1, one of the miR-34a-5p targets, was selected to further validate their direct interaction because down-regulation of Tia1 increases inflammatory response and chronic stress, which may prevent neurogenesis [42,43,44,45]. The luciferase reporter assay was based on the previous description [46]. The fragments of 3′UTR of mouse Tia1 containing the binding sequence of miR34a-5p were amplified by RT-PCR. The primers used were 5′-CAC GAT GGT GGA TGT TTG CC-3′ and 5′-GAT GCG GCG AGG ACT TAT CA-3′. The amplified fragments were directionally cloned into the PmeI and XhoI unique restriction enzyme sites of psiCHECK-2 plasmid (C8021, Promega Corporation, Madison, WI, USA), which are downstream of the Renilla luciferase gene. Transfection efficiency was normalized using firefly luciferase. The miR-34a-5p seed region of Tia1 was mutated using the Phusion Site-Directed Mutagenesis Kit (Thermo Fisher Scientific, Waltham, MA, USA) with primer sequences of 5′ -phosphate ATT CCT TTT TTA AAA ATA AGA GGC-3′ and 5′-phosphate GTC AAT CCC TGC ATT TGT CTT TG. HEK293T cells were co-transfected with 0.2 μg psiCHECK-2 constructed with 3′UTR binding sites of miR-34a-5p and 100 nM miR-34a-5p mimic (Dharmacon, Lafayette, CO, USA) or scrambled mimic control (Dharmacon, Lafayette, CO, USA), using X-tremeGENE siRNA Transfection Reagent (Roche, Basel, Switzerland) according to the manufacturer′ s protocol. The Dual-Luciferase^®^ Reporter Assay System (Promega Corporation, Madison, WI, USA) was used to measure luciferase and renilla signals 48 h after transfection.

### 2.8. qRT-PCR Analysis of Tia1 mRNA

The RNA was reversely transcribed using Maxima first strand cDNA synthesis kits (Thermo Fisher Scientific, Waltham, MA, USA). One microgram of RNA was added to 4 µL 5× Reaction Mix and 2 µL Maxima Enzyme Mix, and topped up to 20 µL with nuclease-free water. Tubes were incubated at 25 °C for 10 min, followed by 50 °C for 45 min and 85 °C for 5 min. The resulting cDNA was then diluted by adding 100 µL of nuclease-free water and stored in aliquots at −20 °C.

Twenty microliters of master mix, for real time PCR, were prepared as follows: 2 µL diluted cDNA, 10 µL 2 x Maxima SYBR Green qPCR Master Mix, 2 µL 10 x forward and reverse primers for target genes, and 4 µL nuclease-free water. The primers were used for Tia1: 5′-GAGAAGGGCTATTCGTTT-3′ and 5′ -CCATACTGTTGTGGGTTT-3′; GAPDH: 5′-GCACCGTCAAGGCTGAGAAC-3′ and 5′-TGGTGAAGACGCCAGTGGA-3′. Samples were denatured at 95 °C for 10 min, followed by 40 cycles of: denaturation at 95 °C for 15 sec, annealing at 60 °C for 30 sec, extension at 72 °C for 30 s PCR amplification was carried out in QuantStudio 6 Real-Time PCR Systems (Thermo Fisher Scientific, Waltham, MA, USA). Fluorescence data were then collected. The expression of GAPDH was used as the internal control.

### 2.9. Western Blot 

The mouse cerebrum was homogenized in CelLytic MT Mammalian Tissue Lysis/Extraction Reagent (Sigma-Aldrich Corporation, St. Louis, MO, USA) containing 100 X Halt™ Protease and Phosphatase Inhibitor Cocktail (Thermo Fisher Scientific, Waltham, MA, USA). The lysate was centrifuged at 15,000× *g* for 15 min, and the supernatant was transferred into a clear Eppendorf tube. Pierce™ BCA Protein Assay Kit (Thermo Fisher Scientific, Waltham, MA, USA) was used to measure protein concentration.

Protein samples were separated by 10% SDS-PAGE, and then transferred to a nitrocellulose membrane. The membrane was blocked by Blotting One (Nacalai Tesque Inc., Kyoto, Japan), incubated with the respective primary antibodies (β-actin, 1:1000, Cell Signalling Technology, Beverly, MA, USA; Tia1, 1:1000, Invitrogen, Thermo Fisher Scientific, Waltham, MA, USA) overnight at 4 °C followed by HRP-conjugated secondary antibodies (1:10,000 dilution) at room temperature for 1h. The WesternBright Sirius Chemiluminescent Detection Kit (Advansta Inc, San Jose, CA, USA) was used to detect immunoreactive proteins. Membranes were then visualized and quantified using the Bio-Rad Gel Doc system. Band densities were measured by ImageJ and normalized by the respective loading control β-actin. The fold change relative to the control was calculated.

### 2.10. Culture of Neural Stem Cells

Mouse cortical neural stem cells (NSCs) were purchased from R&D Systems, Inc. (Minneapolis, MN, USA). Cell culture flasks or plates were pre-coated with matrigel (Gibco, Thermo Fisher Scientific, Waltham, MA, USA). Cells were grown in NeuroCult™ basal medium (STEMCELL Technologies Singapore Pte Ltd., Singapore), and supplemented with NeuroCult™ proliferation supplement (STEMCELL Technologies Singapore Pte Ltd., Singapore), epidermal growth factor (EGF) and basic fibroblast growth factor (bFGF) (Invitrogen, Thermo Fisher Scientific, Waltham, MA, USA) in a humidified incubator at 37 °C with 5% CO_2_. Cells were passaged using accutase (Gibco, Thermo Fisher Scientific, Waltham, MA, USA).

### 2.11. RNA Isolation from NSCs and qRT-PCR Analysis for miR and mRNA

Cells were seeded in a matrigel-coated 6-well plate. RNA isolation and qRT-PCR analysis for miR and mRNA were performed as mentioned above.

### 2.12. Western Blot for NSCs

Cells were harvested in CelLytic MT Mammalian Tissue Lysis/Extraction Reagent (Sigma-Aldrich Corporation, St. Louis, MO, USA) containing 100× Halt™ Protease and Phosphatase Inhibitor Cocktail (Thermo Fisher Scientific, Waltham, MA, USA), and lysed by violent vortex several times before incubating on ice for 20 min on a shaker. After centrifugation at 15,000× *g* for 15 min, the supernatant was transferred into a clear Eppendorf tube. The protein concentration was measured and samples were separated as described above.

### 2.13. Overexpression of Tia1 in NSCs

Mammalian vector pCMV6-AC-GFP, containing Tia1 (NM_009383) Mouse Tagged ORF Clone (No: MG226372, OriGene Technologies, Inc., Rockville, MD, USA) or blank control pCMV6-AC-GFP with C-terminal tGFP tag (No: PS100010, OriGene Technologies, Inc., Rockville, MD, USA), was transfected into NSCs using Lipofectamine 3000 reagent (Thermo Fisher Scientific, Waltham, MA, USA) according to the manufacturer′ s instructions. 

### 2.14. Statistical Analysis

Data were expressed as mean ± SEM, and *p* < 0.05 was considered as statistically significant. The Student’s t-test was used to compare two sets of quantitative data. For the comparison among three or more groups, one-way analysis of variance (ANOVA) was used followed by Bonferroni-corrected pairwise post-hoc tests.

## 3. Results

### 3.1. ϒ-Irradiation at P3 Induced Depression-Like Behavior in Adult Mice 

**Figure 1:**
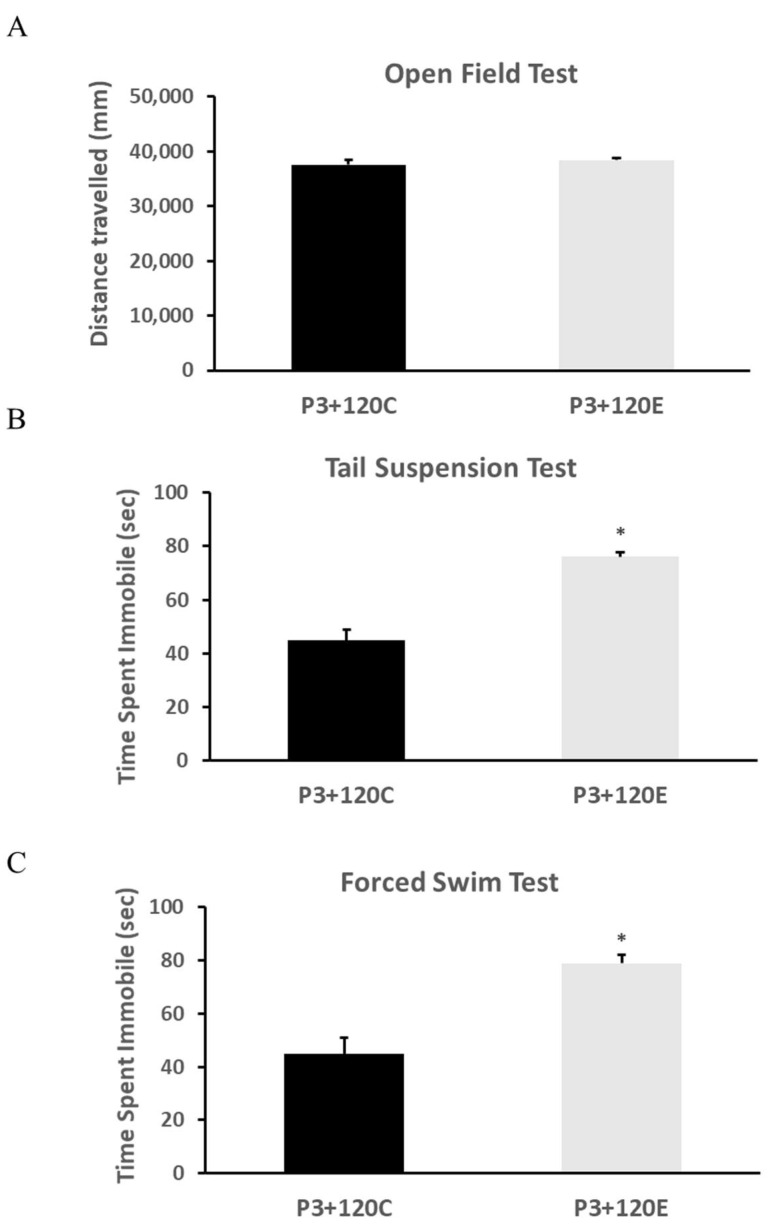
ϒ-irradiation with 5Gy at postnatal day 3 (P3) induced depression-like behavior in adult mice without locomotor activity change. (**A**) The total distance travelled in the open field (locomotor) test; (**B**) the immobile time in the tail suspension test; (**C**) the immobile time in the forced swim test. Data are expressed as mean ± SEM, and analyzed by Student’s *t*-test (*n* = 12). * *p* < 0.05 vs. P3+120C.

The open-field test showed no difference in distance travelled between non-irradiated and irradiated mice (Figure 1A). Irradiated mice displayed a significantly increased time of immobilization in the tail suspension test (Figure 1B) and the forced swim test (Figure 1C) compared to the control, suggesting that γ –irradiation-induced depression-like behavior in adult mice.

### 3.2. ϒ-Irradiation with 5Gy Induced Hypoplasia of The Infrapyramidal Blade of The Stratum Granulosum, and Aberrant and Impaired Neurogenesis in the Subgranular Zone of the Dentate Gyrus

**Figure 2:**
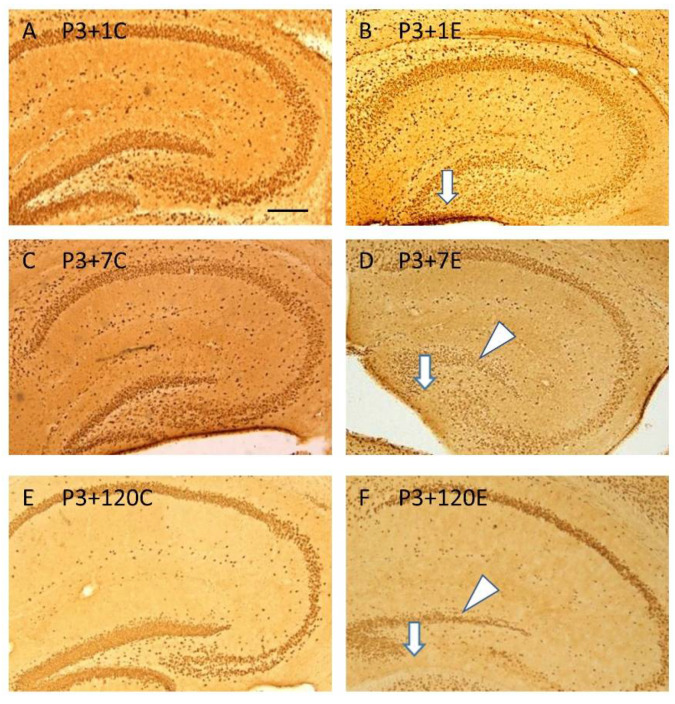
NeuN immunostaining shows that γ-irradiation with 5Gy at postnatal day 3 induced hypoplasia of the infrapyramidal blade of the stratum granulosum of the dentate gyrus (arrows) at 1 ((**B**) compared to (**A**) in the control), 7 ((**D**) compared to (**C**) in the control), and 120 ((**F**) compared to € in the control) days after radiation exposure. Loss of NeuN immunopositive neurons also occurred in the suprapyramidal blade of the stratum granulosum of the dentate gyrus, particularly at 7 (arrowhead in (**D**)) and 120 (arrowhead in (**D**)) days after irradiation when compared to the age-matched control mice (**C**,**E**) (*n* = 5). Scale bar = 200 µm in A applies to (**B**–**F**).

NeuN immunohistochemistry revealed hypoplasia of the infrapyramidal blade of the stratum granulosum of the dentate gyrus, which appeared at 1 day (Figure 2A,B) after irradiation at P3, and persisted from 7 (Figure 2C,D) to 120 days (Figure 2E,F) after irradiation. In the suprapyramidal blade of the stratum granulosum of the dentate gyrus, the loss of NeuN immunopositive neurons also occurred (Figure 2 B,D,F), particularly at 120 days after irradiation (Figure 2F), compared to the age-matched control (Figure 2A,C,E).

**Figure 3:**
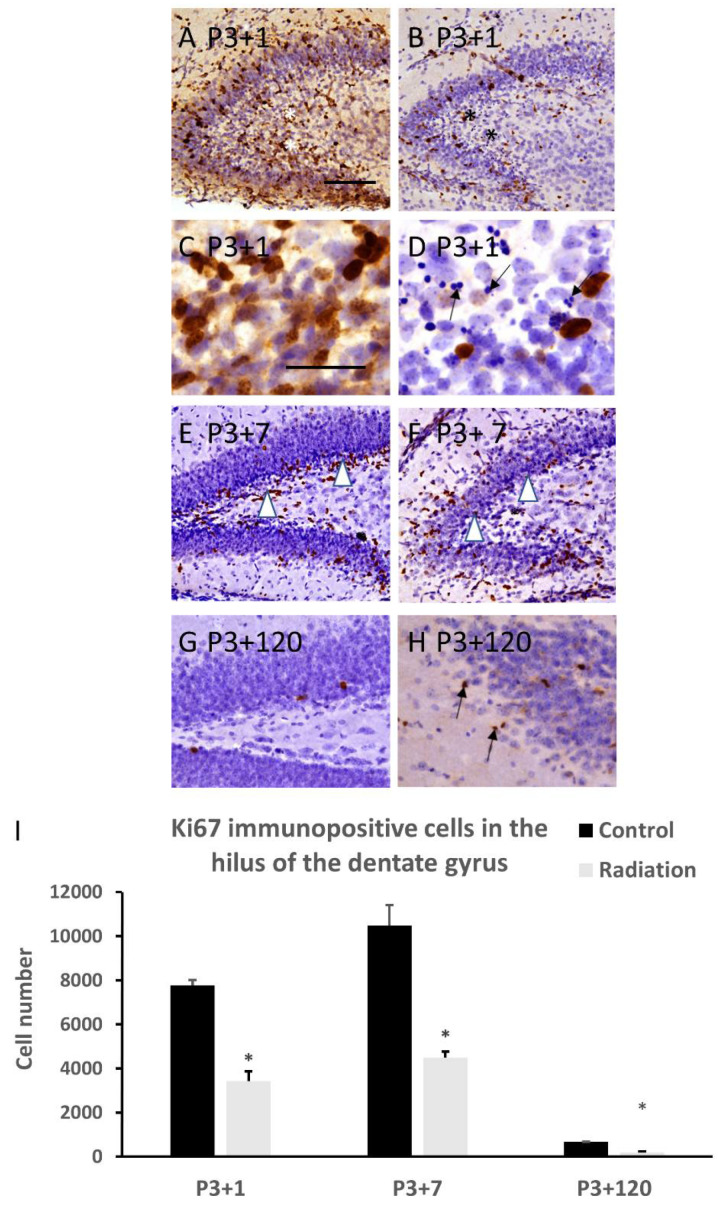
Ki67 immunostaining shows that γ-irradiation with 5Gy at postnatal day 3 induced a significant reduction in Ki67 immunopositive cells in the hilus of the dentate gyrus, including the subgranular zone (asterisks), at 1 (**B**,**D** compared to (**A**), (**C**) in the control, (**C**,**D**) are magnified figures from (**A**,**B**), respectively, (**I**)) (arrows in (**D**) indicate apoptotic bodies), 7 (**F** compared to (**E**) in the control, white arrowheads indicate Ki67 immunopositive cells, (**I**)), and 120 ((**H**) compared to (**G**) in the control, **I**) days after radiation exposure. Aberrant Ki67 immunopositive cells appeared in the molecular layer of the dentate gyrus at 120 days after radiation exposure (arrow in (**H**)) (*n* = 5). * *p* < 0.05 vs. respective control. Scale bar =100 µm in (**A**) applies to (**B**,**E**–**H**). Scale bar = 50 µm in C applies to (**D**).

Ki67 immunohistochemistry indicated a significant reduction in Ki67 immunopositive cells in the hilus, including the subgranular zone of the dentate gyrus, which started at 1 day (Figure 3A–D,I) after irradiation of P3 mice, and persisted from 7 days (Figure 3E,F,I) to 120 days (Figure 3G and H, I) after irradiation exposure. Counterstaining with hematoxyin showed many apoptotic bodies in the hilus (including the subgranular zone) of the dentate gyrus at 1 day after irradiation (Figure 3D). At 120 days after irradiation, aberrant Ki67 immunopositive dividing cells were observed in the molecular layer of the dentate gyrus (Figure 3H).

**Figure 4:**
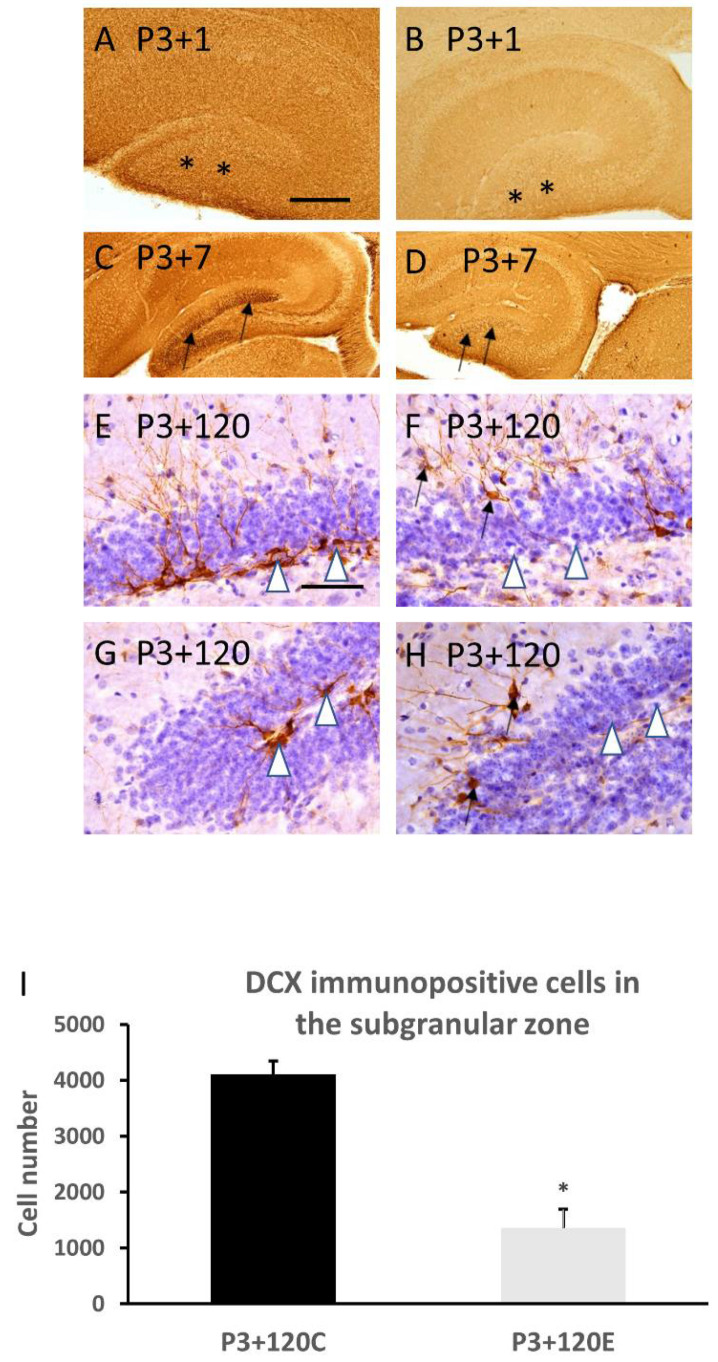
DCX immunostaining shows that γ-irradiation with 5Gy at postnatal day 3 induced an obvious reduction in DCX immunopositive product in the dentate gyrus at 1 day ((**B**) compared to (**A**) in the control, asterisks) and 7 days ((**D**) compared to (**C**) in the control, in particular in the subgranular zone, arrows indicate DCX immunopositive cells). Quantitative study indicates a significant reduction in DCX immunopositive neurons in the subgranular zone of the dentate gyrus at 120 days after radiation exposure (white arrowheads in (**F**), (**H**) indicate subgranular zone with fewer DCX immunopositive cells compared to those in (**E**,**G**) which indicate more DCX immunopositive cells. (**I**)). Aberrant DCX immunopositive neurons also appeared in the molecular layer of the dentate gyrus at 120 days after radiation exposure (arrows in (**F**,**H**)) (*n* = 5). * *p* < 0.05 vs P3+120C. Scale bar =200 µm in (**A**) applies to (**B**–**D**). Scale bar = 100 µm in E applies to (**F**–**H**).

DCX immunohistochemistry showed a homogenous distribution of DCX in the dentate gyrus at postnatal day 4 (P3+1) (Figure 4A) and day 10 (P3+7) (Figure 4C) in the control (Figure 4A,C) and experimental (Figure 4B,D) mice. In the experimental mice (Figure 4B,D), DCX immunostaining in the entire dentate gyrus was weaker than in the control mice (Figure 4A,C), particularly in the subgranular zone at 7 days after irradiation (Figure 4D compared to Figure 4C). At 120 days after irradiation, the number of DCX immunopositive cells in the subgranular zone of the dentate gyrus was significantly reduced in the irradiated mice (Figure 4F,H,I) compared to the control (Figure 4E,G). Furthermore, aberrant newly generated DCX immunopositive cells were observed in the molecular layer of the dentate gyrus (Figure 4F,H).

### 3.3. Systematic miRSeq and Real Time RT-PCR

**Figure 5:**
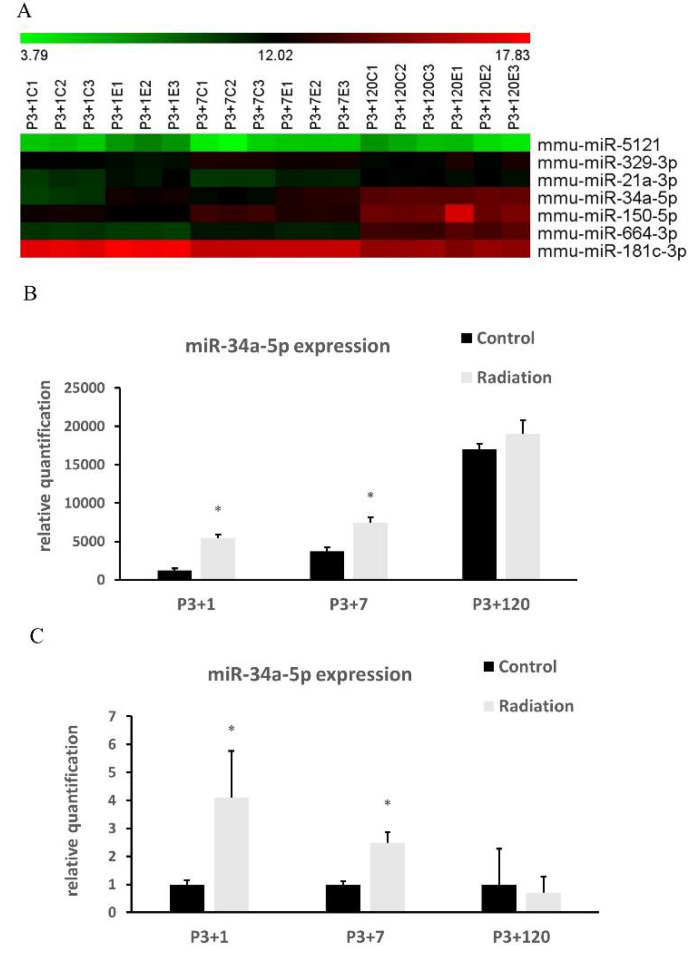
A heatmap displays seven differentially expressed miRNAs identified by systematic deep microRNA sequencing (miRSeq) among mice at 1, 7, 120 day(s) after irradiation at P3 and the respective control (**A**). The color bar from left to right represents the expression levels from low to high, and the number indicates the base-2 logarithm of miR expression. Among these miRNAs, the expression of miR-34a-5p is summarized in (**B**). Real-Time Quantitative Reverse Transcription PCR (qRT-PCR) validates the increased expression of miR-34a-5p at 1 and 7 day(s) after radiation exposure (**C**). Data are expressed as mean ± SEM (*n* = 3). * *p* < 0.05 vs respective control.

A total of 771 miRs was detected and analyzed. Seven miRs with significant changes between control and irradiated mice at P3+1 and P3+7 groups were summarized by the heatmap (Figure 5A). Statistical analysis indicated upregulation of miR-34a-5p at 1 and 7 day(s), but not 120 days after irradiation (Figure 5B). qRT-PCR further validated miRSeq and showed a significantly increased expression of miR-34a-5p in the mouse brain at 1 and 7 day(s) after irradiation (Figure 5C).

### 3.4. miR-34a-5p Targeted on mRNA of Tia1

**Figure 6:**
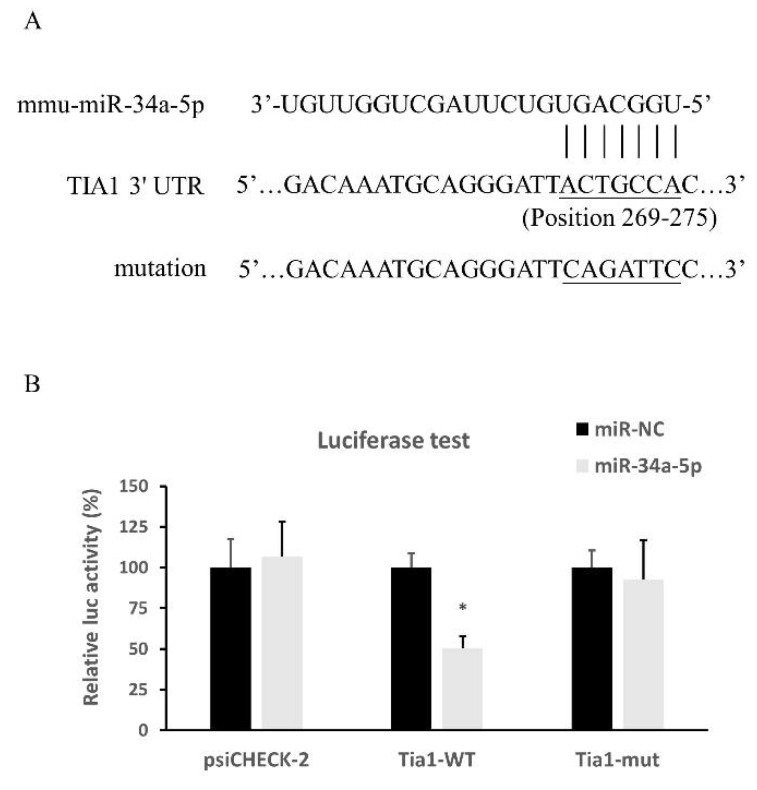
Direction interaction of miR-34a-5p with Tia1 examined by luciferase reporter assay. (**A**) Sequence alignment of putative miR-34a-5p binding sites in Tia1 3ʹ UTRs, and the mutation sequences. (**B**) Activity of luciferase gene linked to the 3’ UTR of Tia1 mRNA. HEK293T cells were co-transfected with psiCHECK-2 constructed with 3′ UTR binding sites of miR-34a-5p and miR-34a-5p mimic or scrambled mimic control. Luciferase and renilla signals were measured 48 h after transfection. Data are expressed as mean ± SEM. * *p* < 0.05.

The binding site of mouse miR-34a-5p exists in the position 269 to 275 of Tia1 3′ UTR (Figure 6A), and their direct interaction, was confirmed by luciferase reporter assay (Figure 6B). 

No change in luciferase activity was observed when the miR-negative control (miR-NC) or miR-34a-5p mimic was transfected into the blank plasmid psiCHECK-2 (Figure 6B). However, when miR-34a-5p mimic was co-transfected with psiCHECK-2 containing mouse Tia1 3′ -UTR binding sequence into HEK 293 cells, the luciferase intensity was reduced significantly compared to the negative control (Figure 6B), indicating the binding of the miR-34a-5p and 3′ -UTR regions of Tia1. miR-34a-5p did not bind to the mutant Tia1 3′-UTR sites (Figure 6B), suggesting that Tia1 may function as the direct target of miR-34a-5p.

### 3.5. mRNA and Protein Expression of Tia1 in Mice Brain after Γ-Irradiation with 5Gy 

**Figure 7:**
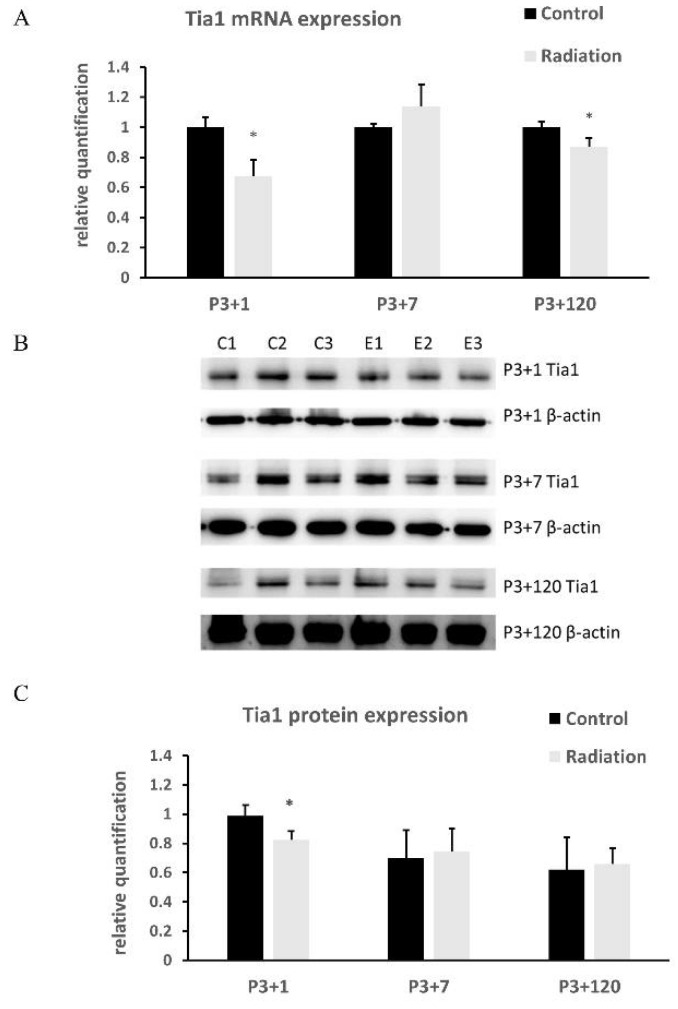
The expression of Tia1 in animal brains. Balb/c mice were γ-irradiated with 5Gy at P3, and brain samples were collected at 1, 7, and 120 day(s) after irradiation. (**A**) mRNA expression of Tia1 by qRT-PCR; (**B**) western blot images of Tia1; (**C**) statistical results of Tia1 protein levels. Data are expressed as mean ± SEM (n = 3). * *p* < 0.05 vs respective control.

Tia1 mRNA decreased significantly at 1 and 120 day(s), but not at 7 days, after γ-irradiation with 5Gy compared to the respective controls (Figure 7A). A significant decrease in Tia1 protein occurred at 1 day (Figure 7B,C), but not 7 or 120 days (Figure 7B,C), after γ-irradiation.

### 3.6. Γ-Irradiation Increased the Expression of miR-34a-5p in NSCs and Decreased the mRNA Expression of Tia1

**Figure 8:**
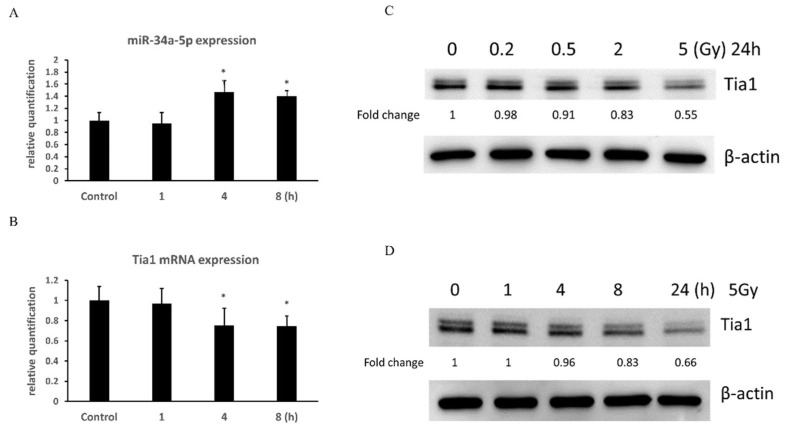
The expression of miR-34a-5p and Tia1 in NSCs after γ-irradiation. Time-dependent change of miR-34a-5p (**A**) and Tia1 (**B**) mRNA expression in NSCs after γ-irradiation with 5Gy. Data are expressed as mean ± SEM (n = 3). * *p* < 0.05 vs control. The protein expression of Tia1 in NSCs examined by western blot is dose- (**C**) and time-dependent (**D**) after γ-irradiation.

qRT-PCR indicated the increased expression of miR-34a-5p (Figure 8A) and decreased Tia1 miRNA (Figure 8B) in NSCs from 4 to 8h, but not at 1h after γ-irradiation with 5Gy. This negative correlation between the expression of miR and its target mRNA further confirms that Tia1 is a direct target of miR-34a-5p.

### 3.7. Γ-Irradiation Dose- and Time-Dependently Decreased Protein Expression of Tia1 in NSCs 

NSCs were γ-irradiated with different doses from 0.2 to 5Gy. Western blot results showed that the γ-irradiation dose-dependently decreased the protein levels of Tia1 in NSCs (Figure 8C). When NSCs were γ -irradiated with 5 Gy, the reduction in Tia1 protein levels was time-dependent (Figure 8D).

### 3.8. Overexpression of Tia1 Partially Blocked Γ-Irradiation-Induced Impairment of Neurogenesis

**Figure 9:**
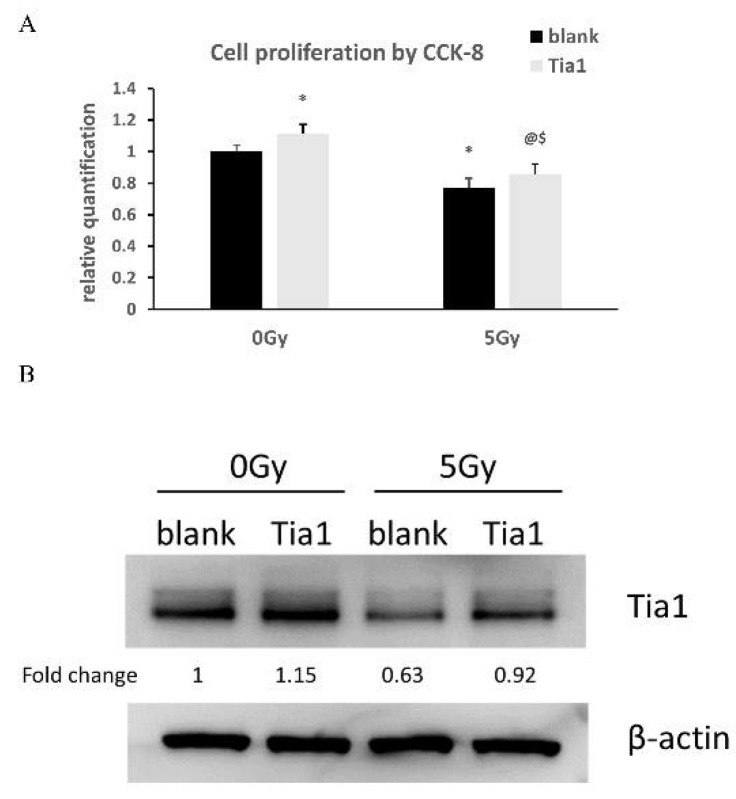
Effects of overexpression of Tia1 in NSCs after γ-irradiation. pCMV6-AC-GFP containing Tia1 or blank control was transfected into NSCs, which were then γ-irradiated with 5Gy or not irradiated as a control. (**A**) Cell proliferation in transfected NSCs. Data are expressed as mean ± SEM (*n* = 5). * *p* < 0.05 vs blank 0 Gy; @ *p* < 0.05 vs. Tia1 0 Gy; $ *p* < 0.05 vs. blank 5 Gy. (**B**) Protein expression of Tia1 in transfected NSCs examined by western blot.

Γ-irradiation significantly decreased cell proliferation in NSCs transfected with either blank or Tia1 plasmid when compared with the respective non-irradiated groups (Figure 9A). The transfection of Tia1 plasmid partially blocked the loss of proliferating cells induced by γ-irradiation in NSCs (Figure 9A). The overexpression of Tia1 also enhanced cell proliferation in the non-irradiated groups as compared to NSCs transfected with blank plasmid (Figure 9A). 

Western blotting showed that the transfection of Tia1 into NSCs enhanced the Tia1 protein levels compared to the blank plasmid group (Figure 9B). γ-irradiation with 5Gy reduced the Tia1 protein levels in NSCs transfected with blank plasmid, while this decrease was reversed by the transfection of Tia1 plasmid (Figure 9B), suggesting that promoting Tia1 expression in brain neurogenesis niches may be a novel therapeutic approach to prevent the radiation-induced impairment of neurogenesis.

## 4. Discussion

### 4.1. Radiation, Hippocampal Neuropathology and Depression

Survivors of major nuclear accidents or war may experience brain damage and depression [1,2,47,48]. In Chernobyl clean-up workers and liquidators with depression, the radiation-induced dysfunction of the cortico-limbic system in the left dominant hemisphere of the brain, with a specific involvement of the hippocampus, is considered to be the key cerebral basis of post-radiation brain damage [1,2,47,48]. Patients with depressive symptoms have smaller dentate gyrus [5]. This was supported by a recent study showing that early life adversity increased major depressive disorder (MDD) and suicide risk and could potentially affect the dentate gyrus, leading to a smaller dentate gyrus and fewer granular neurons in MDD [12]. In the present study, irradiation at P3 induced depression in adult animals. A neuropathological study showed that irradiation-induced hypoplasia of the infrapyramidal blade of the stratum granulosum of the dentate gyrus and neuronal loss occurred as early as one day after irradiation. At 120 days after irradiation, hypoplasia of the infrapyramidal blade of the stratum granulosum of the dentate gyrus persisted; meanwhile, aberrant cell division and neurogenesis in the molecular layer of the dentate gyrus were found. Our animal study strongly supports the conclusion from human data [12] and suggests that early life radiation-induced hypoplasia of the infrapyramidal blade of the stratum granulosum, as well as impairment and aberrant neurogenesis of the dentate gyrus, may be related to smaller dentate gyrus sizes and the development of depression. These pathological changes also support the neurogenesis and cellular plasticity hypotheses of depression. Radiation was shown to induce depression in mouse models [17,18,19,20]. However, previous studies on adult animals did not show hypoplasia of the infrapyramidal blade of the stratum granulosum of the dentate gyrus and aberrant cell division and neurogenesis. The pathological changes at 1 day after irradiation of P3 mice strongly suggest that therapeutic approaches to prevent early life adversity may impede the development of different neuropsychological disorders at the late stages of human life.

### 4.2. miR-34a-5p in Neurogenesis and Depression

Increased miR-34a was detected in the brain, blood and cerebrospinal fluids of patients or animals with depression [28]. Of the differentially expressed miRs in the present study, we chose to validate miR-34a-5p and investigate its target due to the fact that it negatively regulates neural stem cell proliferation, differentiation, neuronal migration and maturation [49,50,51]. Brain miR-34a-5p changes were also found to be involved in other brain insults [52]. Radiation-induced miR-34a-5p changes in the blood may be involved in the development of Alzheimer′ s disease, depression and schizophrenia, suggesting that miR-34a-5p may be involved in the common brain neuropathological changes in these diseases, such as impairment of neurogenesis [28]. Altered miR-34a-5p in the cerebrospinal fluid and blood was considered as an early biomarker of major depression disorder [53] or Alzheimer’s disease [54]. Plasma miR-34a-5p expression may be used to distinguish radiation exposure levels in mice [55]. Furthermore, miR-34a-5p contributes to synaptic plasticity via dis-inhibition of the translation of key plasticity-related molecules [56]. The upregulation of miR-34a-5p was revealed at 1 and 7 day(s) after irradiation at P3 in the present study. Radiation-induced upregulation of miR-34a-5p and reduced cell proliferation were further confirmed in a neural stem cell model. miR-34a is a tumor-suppressor gene and overexpression of miR-34a suppressed the expression of 136 neuronal progenitor genes [57]. The radiation-induced upregulation of brain miR-34a-5p in both animal and stem cell models may prevent brain neurogenesis, leading to hypoplasia of the infrapyramidal blade of the stratum granulosum, impairment of neurogenesis, aberrant cell division and neurogenesis in the molecular layer of the dentate gyrus, and subsequent depression.

### 4.3. miR-34a-5p Targets Tia1 to Prevent Neurogenesis 

miR-34a targets E2F3, Numbl, platelet-derived growth factor receptor A (PDGFRA), synaptotagmin 1 (Syt1), autophagy-related 9a (Atg9a), CD44, cyclic AMP response-element binding protein (CREB), brain-derived neurotrophic factor (BDNF) to inhibit cell proliferation, migration, invasion and adhesion [58,59,60,61,62,63,64,65]. It regulates extracellular signal-regulated kinase 1/2 (ERK) and p38, and increases nuclear factor kappa light chain enhancer of activated B cells (NF-κB), Sirtuin 1 (SIRT1) and Bcl2, to promote or reduce apoptosis [66,67,68]. The miR-34a- A central nucleotide-binding oligomerization domain (NOD)-, C-terminal leucine rich repeat domain (*LRR*)– and caspase activation and recruitment domains (CARDs) -containing 5 (NLRC5)

-NFκB signaling pathway may be involved in HIV-1 Tat-mediated microglial inflammation [69]. miR-34a-5p suppresses tumorigenesis by targeting the Notch signalling pathway [70] or high mobility group AT-hook 2 (HMGA2) [71]. It inhibited proliferation, migration, and cell invasion, accompanied by targeting of matrix metalloproteinase 9 (MMP9) activity and the microtubule-associated protein 2 (MAP2) protein, to reduce their expression [63]. miR-34a-5p also inhibits N-methyl D-aspartate(NMDA) receptors, leading to neuroplasticity changes and Alzheimer’s disease development [72]. Radiation-induced up-regulation of miR-34a-5p in the small intestine and peripheral blood down-regulated hippocampal BDNF, leading to the cognitive impairment [55]. In the present study, we chose Tia1 from many miR-34a-5p targets to confirm its gene and protein expression and validate its direct interaction with miR-34a-5p. This is because Tia1 reduction increases detrimental inflammatory responses in different types of cells and tissues such as bone [44], endometrium [42], peritoneal macrophages [45], and the central nervous system during chronic stress [43]. It controls a large network of immune system genes with modulatory roles in synaptic plasticity and long-term memory, which may be involved in stress-related psychiatric conditions [73]. Tia1 mRNA is detectable in the mouse brain during embryogenesis [74], and is linked to the pathophysiology of neurodegeneration, e.g., Alzheimer′ s disease [39]. Previous studies suggested that Tia1 acts as the target of several miRs, e.g., miR-19a, miR-487a and miR-599 [75,76,77]. In both animal and neural stem cell models, we confirmed that irradiation reduced Tia1 gene and protein expression. In the neural stem cell models, γ-irradiation dose- and time-dependently decreased cell proliferation in NSCs, accompanied by the increase in miR-34a-5p expression as well as the reduction in Tia1 mRNA and protein expression. The luciferase reporter assay indicated a direct interaction between miR-34a-5p and Tia1. The overexpression of Tia1 in NSCs partially reversed the decrease in cell proliferation induced by γ-irradiation, strongly suggesting the involvement of Tia1 in neurogenesis. Our results, therefore, suggest that the radiation-induced interaction of miR-34a-5p and Tia1 may increase inflammatory responses of neural stem cells, leading to the impairment of neurogenesis and the subsequent hypoplasia of the infrapyramidal blade of the stratum granulosum, and aberrant cell division and neurogenesis in the molecular layer of the dentate gyrus.

## 5. Conclusions

The present study showed that irradiation in P3 mice induced hypoplasia of the infrapyramidal blade of the stratum granulosum, and aberrant and impaired cell division and neurogenesis in the dentate gyrus. Hypoplasia of the infrapyramidal blade of the stratum granulosum and impairment of cell division and neurogenesis in the dentate gyrus may be related to the radiation-induced upregulation of miR-34a-5p and downregulation of its target gene Tia1. Our results may provide new clues for understanding the mechanism of the development of depression and for the development of novel therapeutic approaches to the prevention or treatment of depression.

## Data Availability

The data presented in this study are available on request from the corresponding author. The data are not publicly available due to ethical restrictions.

## Abbreviations

ANOVA	Analysis of variance
Atg9a	Autophagy-related 9a
BDNF	Brain-derived neurotrophic factor
bFGF	Basic fibroblast growth factor
CREB	Cyclic AMP response-element binding protein
DCX	Doublecortin
EGF	Epidermal growth factor
ERK	Extracellular signal-regulated kinase ½
HEK	Human embryonic kidney
HMGA2	High Mobility Group AT-Hook 2)
MAP2	Activity and microtubule-associated protein 2
MDD	Major depressive disorder
miR	MicroRNA
miRSeq	MicroRNA sequencing
MMP9	Matrix metalloproteinase 9
NF-κB	Nuclear factor kappa light chain enhancer of activated B
NLRC5	A central nucleotide-binding oligomerization domain (NOD)-, C-terminal leucine rich repeat domain (LRR)- and caspase activation and recruitment domains (CARDs) -containing 5
NMDA	N-methyl D-aspartate
NUMBL	NUMB Like Endocytic Adaptor Protein
NSC	Neural stem cell
PDGFRA	Platelet-derived growth factor receptor A
qRT-PCR	Real-time quantitative reverse transcription polymerase chain reaction
SEM	Standard error of the mean
SIRT1	Sirtuin 1
Syt1	Synaptotagmin 1
Tia1	T-cell intracytoplasmic antigen-1
UTR	Untranslated region
